# A Rare Case of Disseminated Coccidioidomycosis Presenting as Brachial Plexopathy

**DOI:** 10.7759/cureus.11370

**Published:** 2020-11-07

**Authors:** Rahul Shah, Elena Shanina, Robert G Smith, Anish Bhardwaj

**Affiliations:** 1 Neurology/Neurocritical Care, Bakersfield Memorial Hospital, Bakersfield, USA; 2 Neurology, University of Texas Medical Branch, Galveston, USA; 3 Neurology, Houston Methodist Hospital, Houston, USA

**Keywords:** coccidioidomycosis, brachial plexopathy, peripheral nervous system, electromyography and electro-stimulation, neuromuscular ultrasound, adult neurology, fungal infections, cns infection, disseminated coccidioides

## Abstract

Coccidioidomycosis, a fungal infection caused by inhaling spores of *Coccidioides immitis*/*posadasii*, is endemic to the southwestern states of the United States, Northern Mexico and some parts of Central and South America. It is primarily a pulmonary infection with less than 0.5% of symptomatic cases showing dissemination. Skin, lymph nodes and bone are the commonest sites. Neurological involvement is rare and commonly presents as strokes, abscesses or meningoencephalitis. We present the case of a previously healthy 23-year-old African American male, presented with a four-month history of progressive right upper extremity weakness that initially started with right shoulder pain followed by worsening weakness and loss of muscle mass. Electromyography (EMG) demonstrated right brachial plexopathy with moderate-to-severe active denervation changes. MRI cervical spine revealed a 9-cm contrast enhancing extradural mass extending through the neural foramen from C4-T1 roots and forming a 4-cm right apical lung mass subsequently seen on MRI of the brachial plexus. All trunks, divisions and cords were thickened, hyperintense and showed contrast enhancement on MRI. Neuromuscular ultrasound (NUS) demonstrated enlargement of peripheral nerves. Differentials prior to biopsy of the mass ranged from neurofibromas to pancoast lung tumors. Coccidioidomycosis did not figure on the initial list of differentials. Patient underwent subsequent biopsy of the extradural and lung masses that showed coccidiodes. Serum coccidioides antibody titers were elevated. The patient was treated with high-dose intravenous fluconazole and aggressive mass debridement. His weakness improved on four months follow-up evaluation with significant resolution of EMG abnormalities and decreased swelling on NUS.

## Introduction

Coccidioidomycosis, a fungal infection caused by inhaling spores of *Coccidioides immitis*/*posadasii*, is endemic to the southwestern states of the United States, Northern Mexico and some parts of Central and South America. Although primarily a pulmonary infection, less than 0.5% of symptomatic cases show dissemination. Skin, lymph nodes and bone are the commonest sites [1,2]. Neurological involvement is rare and commonly results in meningitis. Less frequent neurological presentations may be brain abscesses, stroke, hydrocephalus and spinal cord compression. To our knowledge, peripheral nervous system involvement as a presentation has not previously been reported.

## Case presentation

**Figure 1:**
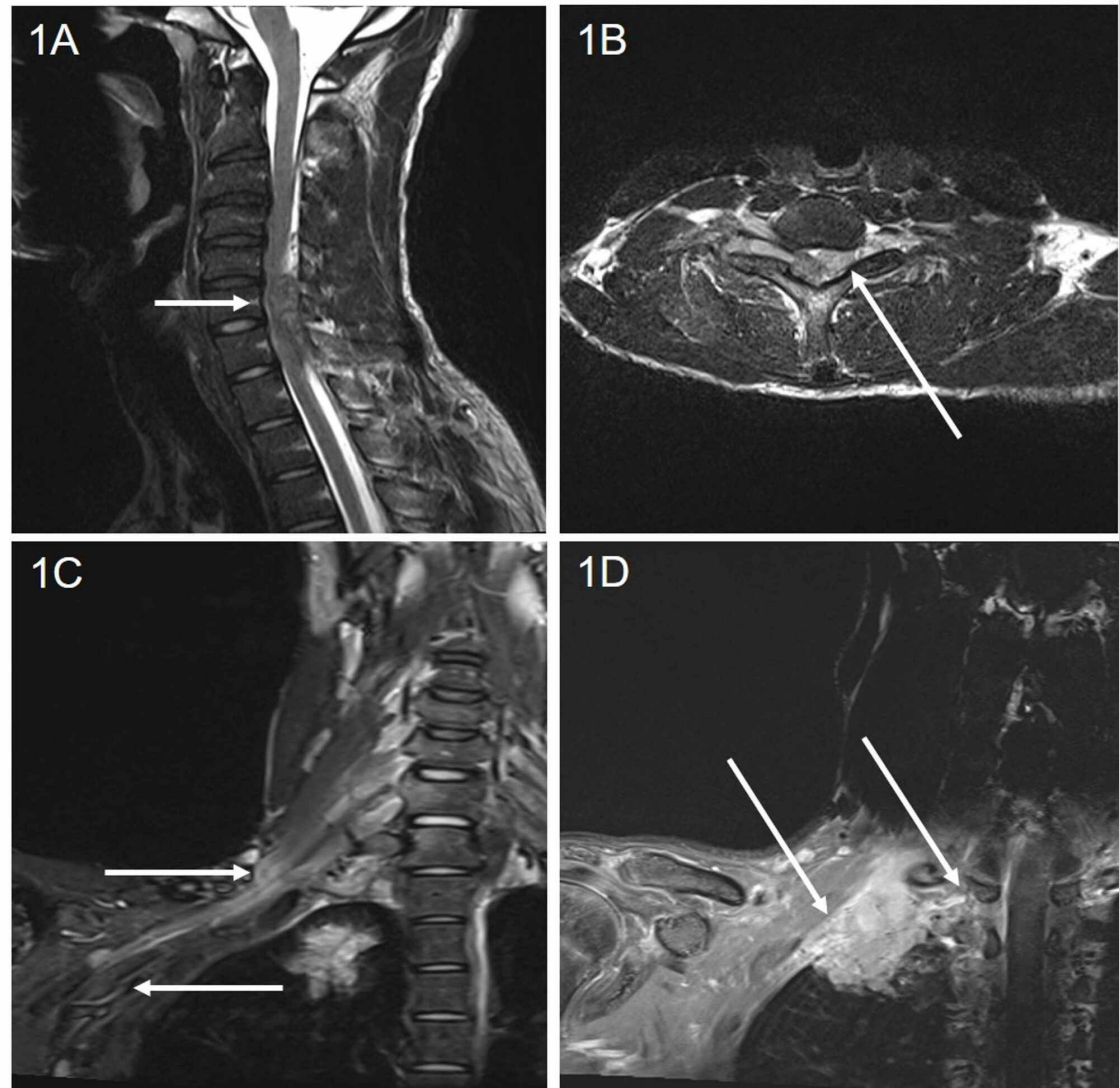
MRI images of cervical spine and right brachial plexus (1A) MRI of the cervical spine - sagittal view of the neck showing large heterogeneous enhancing right-sided extradural mass in the spinal canal extending over a distance of 9 cm and also extending into all right neural foramina from C3-C4 through C7-T1 levels. (1B) MRI of the cervical spine - axial view again showing extradural mass displacing the spinal cord to the left with evidence for osseous remodeling along the mass. (1C) MRI of the right brachial plexus showing thickened and hyperintense trunks, divisions and cords of the right brachial plexus, which probably represent neuritis. (1D) MRI of the right brachial plexus showing abnormal signal in the right transverse processes of C6 and C7 vertebra, and right aspects of C7 and T1 vertebral bodies, consistent with bone involvement.

A previously healthy 23-year-old African American male, an East Texas native, presented with a four-month history of progressive right upper extremity (RUE) weakness. Initial right shoulder pain was followed by rapidly progressive weakness, with inability to raise his right arm above his head. Weakness gradually progressed to involve the entire RUE with loss of proximal muscle mass. History was negative for any other symptoms including fevers, cough, chills, weight loss and bowel or bladder involvement. Examination revealed a well-nourished young male with remarkable atrophy of the right arm and shoulder girdle muscles, 3/5 strength in proximal and distal muscles, altered sensation in the entire RUE and diminished RUE reflexes. Electromyography (EMG) demonstrated right brachial plexopathy with moderate-to-severe active denervation changes (Figure 1).

Brachial plexus and cervical spine MRI revealed a 9-cm contrast enhancing extradural mass extending through the neural foramen from C4-T1 roots and forming a 4-cm right apical lung mass. There was no obvious bone involvement seen. Furthermore, all trunks, divisions and cords of the right brachial plexus were thickened and hyperintense on T2 imaging, with post-contrast enhancement on T1 sequences (Table 1).

**Table 1:** EMG findings EMG: electromyography; INS ACT: insertional activity; FIB: fibrillations; FASC: fasciculations; AMP: amplitude; DUR: duration; POLY: polyphasic units; RECR: recruitment; DISC: discharge; PI: posterior interosseus nerve Up arrow (↑, ↑↑, ↑↑↑, ↑↑↑↑): mildly increased, moderately increased, marked increased, maximally increased, respectively. Down arrow (↓, ↓↓, ↓↓↓, ↓↓↓↓): mildly decreased, moderately decreased, markedly decreased, maximally decreased, respectively. EMG showing active, moderate severity, denervation of all muscles tested in the right upper extremity, thoracic paraspinal and hypoglossus muscles. Nerve conduction study is grossly normal.

Side	Muscle	Nerve	Root	INS ACT	FIB	FAS	AMP	DUR	POLY	RECR	DISC
Right	1st dorsal interosseus	Ulnar	C8-T1	↑	0	0	↑↑	+/-	<15%	↓	0
Right	Pronator teres	Median	C6-7	↑	1+	0	Normal	Normal	<15%	Poor activity	0
Right	Flexor carpi ulnaris	Ulnar	C8-T1	↑	+/-	0	↑↑	↑	<15%	↓↓	0
Right	Extensor carpi radialis longus	Radial	C6-7	↑	2+	0	↑↑↑	↑↑↑	<15%	↓↓↓	0
Right	Extensor carpi ulnaris	Radial (PI)	C7-8	↑	1+	0	↑	↑	15%-30%	↓	0
Right	Extensor indices	Radial (PI)	C7-8	↑	2+	0	↑↑↑	↑↑↑↑	<15%	↓↓↓	0
Right	Biceps	Musculo-cutaneous	C5-6	Normal	0	0	Normal	↑	<15%	↓↓↓	0
Right	Triceps	Radial	C6-8	↑	0	0	↑	Normal	<15%	↓↓	0
Right	Trapezius	Spinal accessory	CN11,C3-4	Normal	0	0	+/-	↑	15%-30%	↓	0
Right	Deltoid	Axillary	C5-6	↑	2+	0	↑	↑↑	<15%	↓↓↓	0
Right	T7 paraspinal	Rami	T7	Normal	0	0	↑	↑↑	15%-30%		0
Right	Rhomboid major	Dorsal scapular	C5	↑	2+	0	Normal	Normal	<15%	Poor activity	0
Right	Hypoglossus	Hypoglossal	CN12	Normal	0	0	↑	↑	<15%	↓	0
Right	C6 paraspinal	Rami	C6	Normal	0	0	Normal	↑	30%-60%		0

Neuromuscular ultrasound (NUS) demonstrated the enlargement of peripheral nerves and their fascicules not only at the ipsilateral brachial plexus but also in the arm. Initial differential diagnoses included inflammatory versus neoplastic processes.

The patient underwent biopsy of the extradural and lung masses, which showed coccidiodes. Serum coccidioides antibody titers were elevated. The patient was treated with high-dose intravenous fluconazole and aggressive mass debridement. His weakness improved at four months' follow-up evaluation with significant resolution of EMG abnormalities and decreased nerve swelling on NUS. However, the patient went on to subsequently develop further dissemination with paraspinal abscesses, vertebral and tarsal osteomyelitis.

## Discussion

Coccidioidomycosis, a fungal infection caused by inhaling spores of *Coccidioides immitis*/*posadasii*, is endemic to the southwestern states of USA, Northern Mexico and some parts of Central and South America [1,2]. Although primarily a pulmonary infection, less than 0.5% of symptomatic cases show dissemination; skin, lymph nodes and bone are the commonest sites [1,2]. Neurological involvement is rare, and commonly results in meningoencephalitis. Less frequent neurological presentations may be brain abscesses, stroke, hydrocephalus, and spinal cord compression. Extrapulmonary involvement is more common in African and Asian ethnicities and immunocompromised individuals [3,4].

The incidence of coccidioidomycosis has been on the rise [5], possibly due to the increasing use of immunosuppressive medications, increasing incidence of diabetes and other immunosuppressive illnesses, etc. Furthermore, not much is known about the pathophysiology involved in neurological manifestations of coccidioidomycosis, with strong inflammatory response or vasculitis as possible mechanisms [4]. Prognosis after neurological involvement is generally poor, even with aggressive anti-fungal therapy, as seen in our case [4,5].

We did an extensive Medline search with the key words “CNS Coccidioidomycosis”, “neurological Coccidioidomycosis” and “spine Coccidioidomycosis”. Out of a total of 223 relevant cases with documented nervous system involvement, 162 patients had meningeal involvement, 64 had intracerebral abscesses, 39 had spinal cord compression by epidural abscess or fungal granulomas and 2 cases had inflammatory transverse myelitis.

Our patient is the first documented case of coccidioidomycosis presenting with a peripheral nervous system involvement in the form of brachial plexopathy. This case helps to demonstrate the wide clinical spectrum of coccidioidomycosis. It delineates the challenge faced in making a diagnosis of a fairly common but not well-understood infection when presented in an atypical way. Although there was improvement in neurological symptoms initially with aggressive therapy, the patient went on to develop further complications.

## Conclusions

This case represents a rare neurological presentation of coccidioidomycosis, not previously reported. Coccidioidomycosis is being termed as “the other great mimicker” with an ever-expanding range of clinical presentations. It must be listed in differential diagnoses of patients with atypical neurological presentations, especially in susceptible populations and in endemic areas, although with growing travel, previously restrictive geographical limitations for certain endemic infections are no longer considered absolute. Furthermore, the case also represents benefits of early intervention, including aggressive surgical resection for treatment of these cases.
